# Attending to Ecological Temporalities Through Phenology

**DOI:** 10.1080/14688417.2026.2735925

**Published:** 2026-09-21

**Authors:** Keili Koppel

**Affiliations:** Design Informatics, University of Edinburgh, Edinburgh, UK

**Keywords:** Temporalities, phenology, noticing, design research, workshop, gardens

## Abstract

Situating phenology within humanities discourse can open up ways of sensing environmental change locally, and in doing so bring nonhuman temporalities into felt experience. As a science of species’ seasonal behaviours, phenology can be understood as a practice already attuned to nonhuman temporalities, tracking the timing of these behaviours over time. More-than-human temporalities are complex, entangled, and increasingly disrupted by human activities, yet these changes often remain difficult to perceive in everyday life, calling for modes of engagement that can make them perceptible beyond scientific contexts. Taking a design research approach, this paper explores how phenological concepts can be reworked through an exploratory workshop in a garden space, where participants used phenology-inspired lenses to sense more-than-human timings and developed recording protocols for observations of temporal interconnections. By connecting phenology with the ‘arts of noticing’ as complementary practices of attention, this approach can broaden how we sense and understand environmental time.

## Introduction

The Anthropocene and ecological discourses have brought to the fore the intricate interconnections between all Earthly beings, showing how climatic and environmental changes are reshaping more-than-human relations (Chakrabarty 2009; Haraway 2016; Latour 2017). Anthropologist Anna Tsing reminds us that ‘each living thing remakes the world through seasonal pulses’ (2015, 21), and life in a multispecies world depends on coordination, which is always time-dependent (Barad 2007). For survival and for possibilities of better ways of living, nonhuman beings (plants, animals, birds, insects, fungi) must continually adjust their behaviours, drawing on different environmental cues to decide how and when to move, grow, feed or rest (van Dooren and Rose 2016). These timing shifts, or ‘time-making projects’ (Tsing 2015, 21), determine whether beings can perform the actions vital to their lives. For many animal-pollinated plants, successful seed depends on flowering coinciding with the activity of pollinators, a migratory bird finds enough food only when its return aligns with the peak in its food supply, and a seed establishes only when it germinates into suitable conditions. However, we often forget to consider these timing needs of those who cannot voice them in languages we understand or fail to notice how nonhuman temporalities shape our own individual routines (Haraway 2008; Ogden, Hall, and Tanita 2013). Political ecologist Jane Bennett’s notion of ‘vital materialism’ offers a useful reminder that ‘all forces and flows (materialities) are or can become lively, affective, signalling’ (2010, 116–17). Such signals require us to learn to perceive them, which means working out what to look and listen for in a particular place, and building this skill through repeated observation over time.

One established way of perceiving the timings of other species is through the scientific practice known as phenology, which records seasonal phenomena and species’ behaviours over the long term: when trees flower, when fruits ripen, when insects appear (see Figure 1 for an example of a phenophase). The longest known annual phenological record comes from Kyoto, where cherry blossom festivals celebrating the first flowering of cherry trees have been held since the ninth century (Primack et al. 2009). Today, phenological data is collected mostly by citizen volunteers through programmes such as the Woodland Trust Nature’s Calendar and iNaturalist, which provide protocols specifying which stages of plant and animal life to record. These phenophases are defined in detail, and recording is usually organised around single species, tracking a particular plant or animal through its seasonal stages. This focus produces precise, comparable data, but it attends less readily to the relationships between species that shape when those stages occur. The Woodland Trust Nature’s Calendar, for example, describes the right time to record ash budburst ‘when the colour of the new green leaves is just visible between the scales of the swollen bud’ (Woodland Trust Nature’s Calendar 2025). To track ecological changes, these events need to be compared across years, which means phenology depends on consistent, long-term observation. Phenological studies show that with rising temperatures in the UK, plants have significantly advanced their leafing, flowering and fruiting times (Amano et al. 2010; Menzel et al. 2006). This affects migratory birds, who often rely on cues from their wintering grounds, meaning that by the time they return to the UK, food sources have already peaked and become harder to find (Both et al. 2009; Thackeray et al. 2010). Phenology offers methods for perceiving these changing ecological phenomena, and its approaches can help us become more aware of temporal issues in our multispecies entanglements (Bastian and Hawitt 2023).

**Figure 1.**
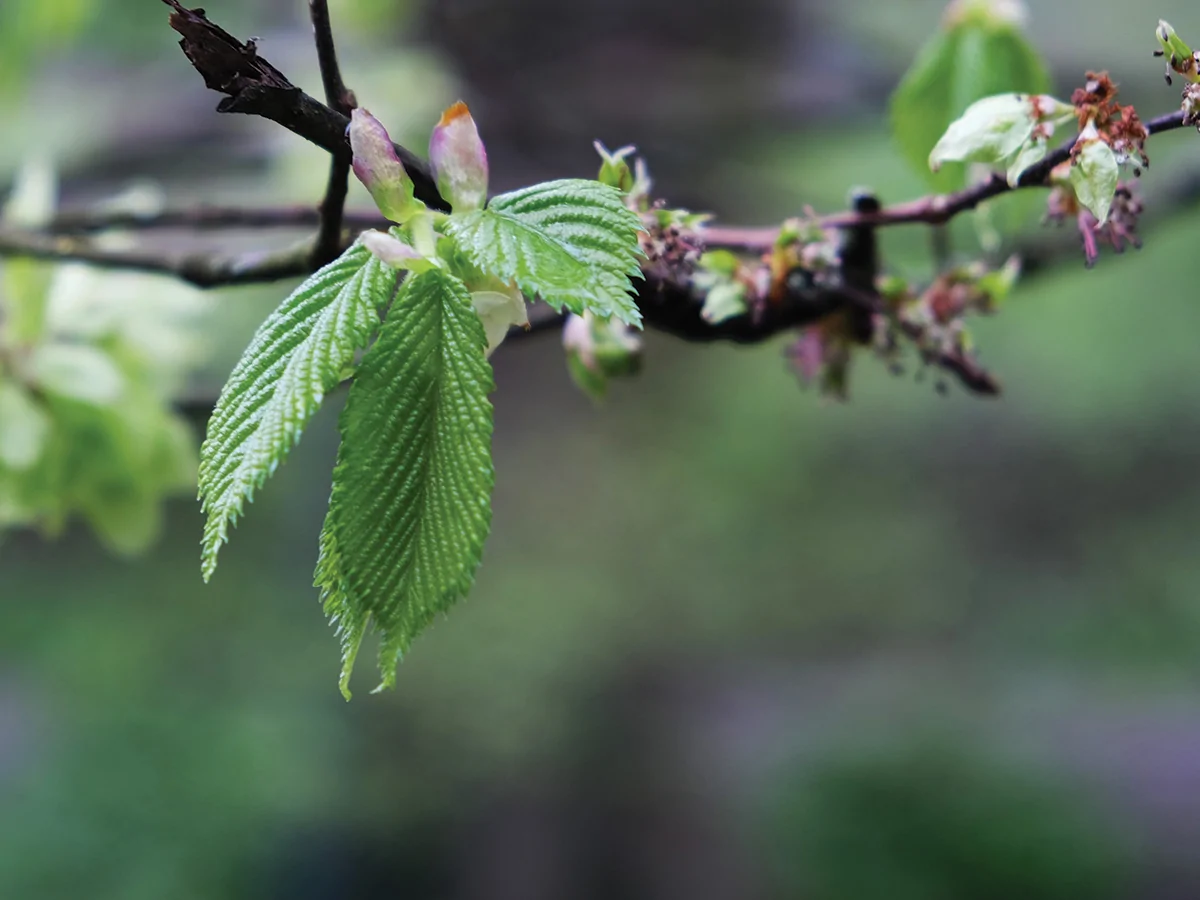
Elm branch showing simultaneous phenophases: open leaves alongside closed and partially opened buds. Photograph by the author.

Seasonal timings have long structured human culture and everyday ways of living, as our consciousness is ‘steeped in a seasonal calendar of nature’s events’ (Macphie and Phillimore 2024, R183). Historically, for example, Ancient Greeks relied on the timing of leaf fall to determine when to sow winter crops (Bostock and Riley 1855). Seasonal events have also carried cultural and emotional significance, such as the arrival of swifts to the UK in spring (Lawrence 2009). With urbanisation, however, seasonal timings have become less visible in shared cultural life, which makes attending to more-than-human timings all the more important for how we collectively make sense of ecological change, including in the stories we tell about it (Clark and Szerszynski 2020; Tsing 2015). This is a concern taken up across environmental humanities and, increasingly, in design research, where attention to multispecies relations is gaining momentum through approaches such as more-than-human care, including in design (Oktay et al. 2024), multispecies ethnography (Elton et al. 2025), multispecies storytelling (Hohti and Tammi 2024; Kirksey and Helmreich 2010; van Dooren and Rose 2016), and temporal design (Pschetz and Bastian 2018). Attending specifically to ecological temporalities within these relations has become a growing concern, with scholars such as Gan (2017) developing frameworks for thinking with nonhuman rhythms and timescales, and Pschetz and Bastian (2018) conceptualising ‘temporal design’ as a way of revealing ‘the intricacies of temporal relationships and negotiations that take place across individuals, groups, and institutions’ (174). Even as these approaches gain traction, the erosion of temporal diversity, the heterogeneity of times and rhythms, remains an urgent concern (Rose 2012).

In this messy process of mutual adjustment, noticing how species affect each other’s time becomes crucial for learning to live attentively within multispecies communities (Rose 2012; Salleh 1997; van Dooren 2014). Without noticing, through small acts of attention, how these timings stand now or how they stood yesterday, it becomes impossible to sense change, and everything risks appearing ordinary (Tsing 2015). We alter others’ times, whether through large-scale climatic effects or the small gestures of daily life: meeting a friend in a park during nesting season or leaving lights on that disrupt nocturnal habits in urban ecologies (Gaston, Visser, and Hölker 2013; Hitch and Leberg 2007). Noticing others’ temporalities, then, becomes a form of ethical and political attention, and a precondition for any kind of repair.

Tsing has noted (Tsing 2024) that noticing is a way of resisting practices that seek to imagine and control the future, drawing our attention instead to the more-than-human world as it exists in the present. By tuning in to the varied timings of the nonhumans around us, we can begin to see how ‘each organism changes everyone’s world’ and to ‘notice the divergent, layered, and conjoined projects that make up worlds’ (Tsing 2015, 22). Through such attention, we can track what Tsing calls ‘unintentional design’, the overlapping world-making activities of many agents, human and otherwise. Tsing (2015) describes this kind of attention through the metaphor of polyphony, the simultaneous act of listening for individual melodies while attending to the harmonies and dissonances they create together (24). This way of listening, she suggests, is what is needed to attune ourselves to the entangled temporalities of humans and nonhumans. Precarity and disturbance become generative ways of thinking within this polyphony, acknowledging the ‘intermittent, spasmodic fruiting’ that reminds us of ‘the precarity of coordination and the curious conjunctures of collaborative survival’ (Tsing 2015, 176). In this project, I take Tsing’s invitation to attend to ‘multiple temporal rhythms’ (2015, 24) quite literally, exploring how concepts from phenological science might open our eyes and ears to the coordinations, interruptions, and entanglements of human and nonhuman lives within the bounded ecology of a garden space.

This paper explores phenology as a humanities practice of attending to species’ timings, describing how phenological concepts were used in a workshop setting to invite participants to observe and record the ecological now around them, who is present at the site and how they appear at a given moment, while being aware of the shifting temporalities in more-than-human life. A single workshop cannot capture the full complexity of a multispecies site, but it can demonstrate an approach. What is described here offers a set of lenses, concepts, and protocols for attending to multispecies sites with time and timings in mind, and for returning to notice what was changed.

## Methodology: lost in West Port Garden Times’ workshop

To explore how to approach the temporalities of a site collaboratively, I held a workshop in late spring 2024 at a small community garden in Edinburgh for people interested in exploring the environment through time (Figure 2), with ethical approval from the Edinburgh College of Art ethics committee. The workshop took a design research approach, developing a set of material and conceptual tools, lenses, a workbook, and a protocol-making activity, through which participants attended to the garden and created their own methods of observation. The approach was exploratory, aimed at opening up what such tools make noticeable. The garden site was selected for its convenience of location (situated in the city centre and open to the public by request) and its compact size, with a dense and readily observable variety of plant and bird species. This configuration was chosen to offer participants immediate points of interest, in contrast to larger urban green spaces where finding different species typically requires more deliberate exploration. To support the exploration of phenology through the humanities, the session was advertised through the Edinburgh Environmental Humanities Network, a university-affiliated network open to broader public membership and therefore directed mostly towards students and staff in humanities courses. Over the two hours, the workshop invited participants to take notice of who was there, to create a reference record for the 6th of June 2024. In addition, by becoming aware of the timings of a given site, participants could develop site-based references for comparing nonhuman behaviours across other habitats (e.g. elsewhere in the UK). The purpose of the workshop was to consider how the entanglements of humans and nonhumans could be tracked and recorded, and to each devise their own methods (protocols) for returning to observe their chosen phenomenon. To begin with, participants had time to explore on their own, then shared their noticings and thoughts in a group discussion towards the end. Questions guiding the exploration were: ‘Whose time is it now? (That is, who is present and how do they appear now?) And how can we notice or attune to more-than-human timings over time?’

**Figure 2.**
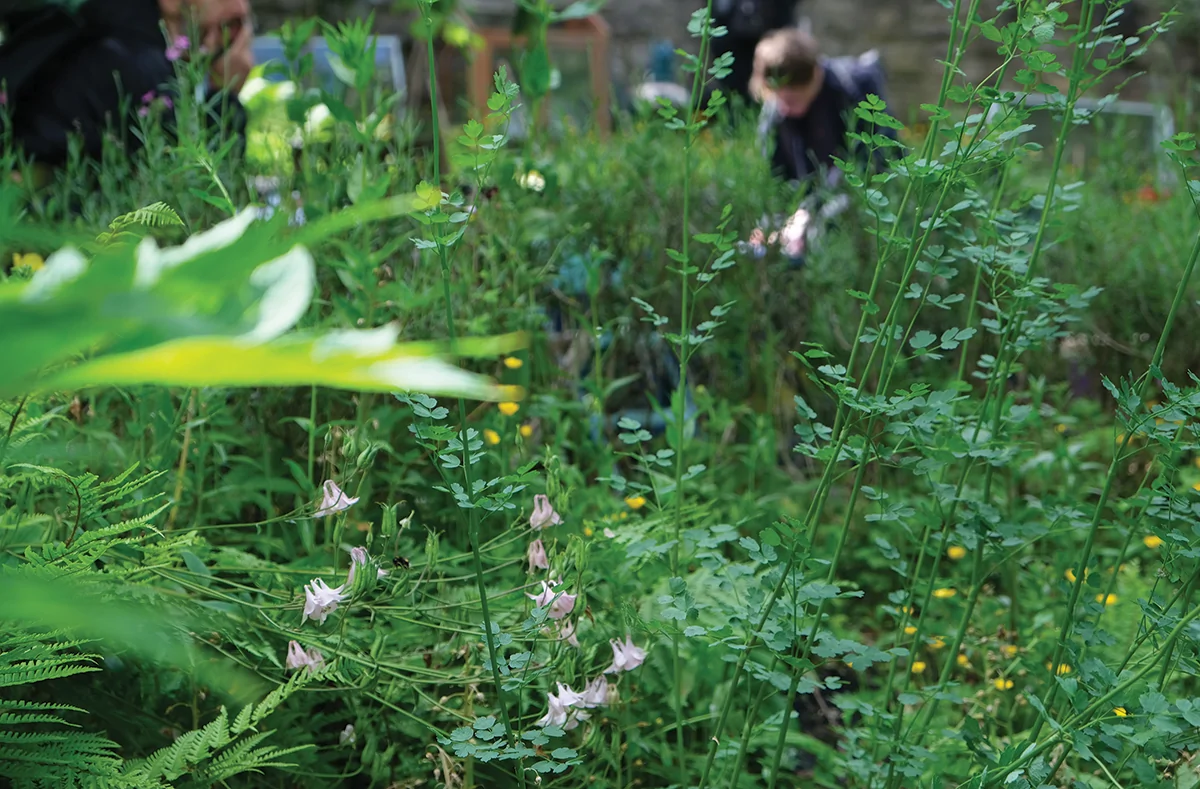
Photo from the workshop run on 6 June 2024 in West Port Garden, Edinburgh, UK. Photograph by the author.

A garden holds many things to notice at once, and it can be hard to know where to begin. To give participants a more concrete way in, each participant was given a lens to focus their attention. These lenses draw on studies in phenology and environmental science that document changing phenomena, mostly linked to climatic changes, and pairs this type of change identified in this research with a mode of sensory attention through which it might be noticed in a garden. These are changes that affect, or could affect, ecological temporal coordination and that might be observable for humans in a garden space over time. More specifically, the ecological-temporal issues can become legible by observing if nonhumans are on time for the social interactions they need to survive and to support other life, and by attending to new or changing phenomena that might be disrupting this coordination. The following lenses are not exhaustive but represent a synthesis based on a number of studies scoped through Google Scholar over two days (by the author). Some focus attention on a mode of sensory perception (colour, sound, touch, movement) and others on a particular taxon (plants and birds).

1. **Colour**. Colour changes in ecologies affect social coordination of nonhumans (e.g. the common example of the snowhare, whose white winter fur becomes a visibility disadvantage when snow cover arrives later due to warming) and leaf-peeping (the cultural practice of visiting sites to observe autumn foliage colour). For example, the loss of colour of bluetits over the past 15-year period studied (López-Idiáquez et al. 2022), the theory that climate change will lead to lighter coloured animals (Delhey et al. 2020), and the brightness of autumn leaf colour caused by heat stress (Koide and Kobayashi 2024).
2. **Plant** (behavioural changes). Warmer springs bring earlier budburst (Vitasse et al. 2022), and pollen seasons are similarly sensitive to spring temperatures: birch pollen release in the UK is strongly correlated with March mean temperature (Newnham et al. 2013). Beyond warming-related shifts, other plant temporalities might be observed, such as the flowers of *Oenothera drummondii* that ‘produce sweeter nectar within 3 min’ after the exposure to the sounds of a flying bee (Karban 2021; Veits et al. 2019), a response whose timing depends on the presence and activity of pollinators but whose own schedules are shifting.
3. **Bird** (behavioural changes). Birds are doing a lot of work to adapt to human environments, e.g. sing with higher frequencies in urban areas to be heard. Silva et al. (2015) also found that species’ dawn and dusk singing developed earlier in the year at sites exposed to light pollution (such as sites near streetlighting or urban centres). There are, however, also limits to what they can adapt to, as Simmonds (2020) reports that ‘Any [bird] population that experienced asynchrony of greater than 24 days with their food source, e.g. caterpillars, went extinct’ (Simmonds 2020).
4. **Sound**. Although useful for detecting the presence of animals, sound data can help us identify the well-being and activities of animals, e.g. Scarpelli et al. (2023) found that ‘nocturnal insects call less frequently on days when the temperature was hotter than average in winter months (June, July, and August)’.
5. **Touch**. Touch can tell us about the qualities of surroundings, such as the temperature or textures of different phenomena. For example, changes in soil texture can be felt as climate warming alters below-ground life, as long-term UK data show steep declines in earthworm abundance and shifts in their seasonal activity linked to soil temperature and moisture (Barnes et al. 2023; Eggleton et al. 2009). These subtle absences make climate change literally tangible beneath our feet. Furthermore, our skin receptors can help us discover subtle changes between phenophases (Pouliot 2023).
6. **Movement**. As water availability shifts across the season, warming and drought reshape how plants move in wind and light. Drought-stressed leaves droop earlier and move with smaller amplitudes, while canopies that once rustled with seasonal regularity may grow still or flutter differently, making plant movement a legible, if overlooked, record of environmental stress (Zhang et al. 2023).

The workshop explored whether these lenses enable participants to attend to the multispecies temporal entanglements (e.g. colour across all the plants and animals in the garden). For each lens, participants were given notes on the relevant scientific studies as a starting point, along with materials specific to the lens (e.g. a sound recorder for sound, coloured pencils for colour).

Through the assigned lens, participants were invited to consider how to capture the changes in this garden space. A workbook was created and shared with each participant to facilitate the garden engagement and exploration through phenological prompts, whilst also assisting the process of note-taking for discussion (see Figure 3–5 for example pages). The workbooks were simply printed and bound, encouraging participants to treat them as working documents to write freely in and mark up as they wished.

**Figure 3.**
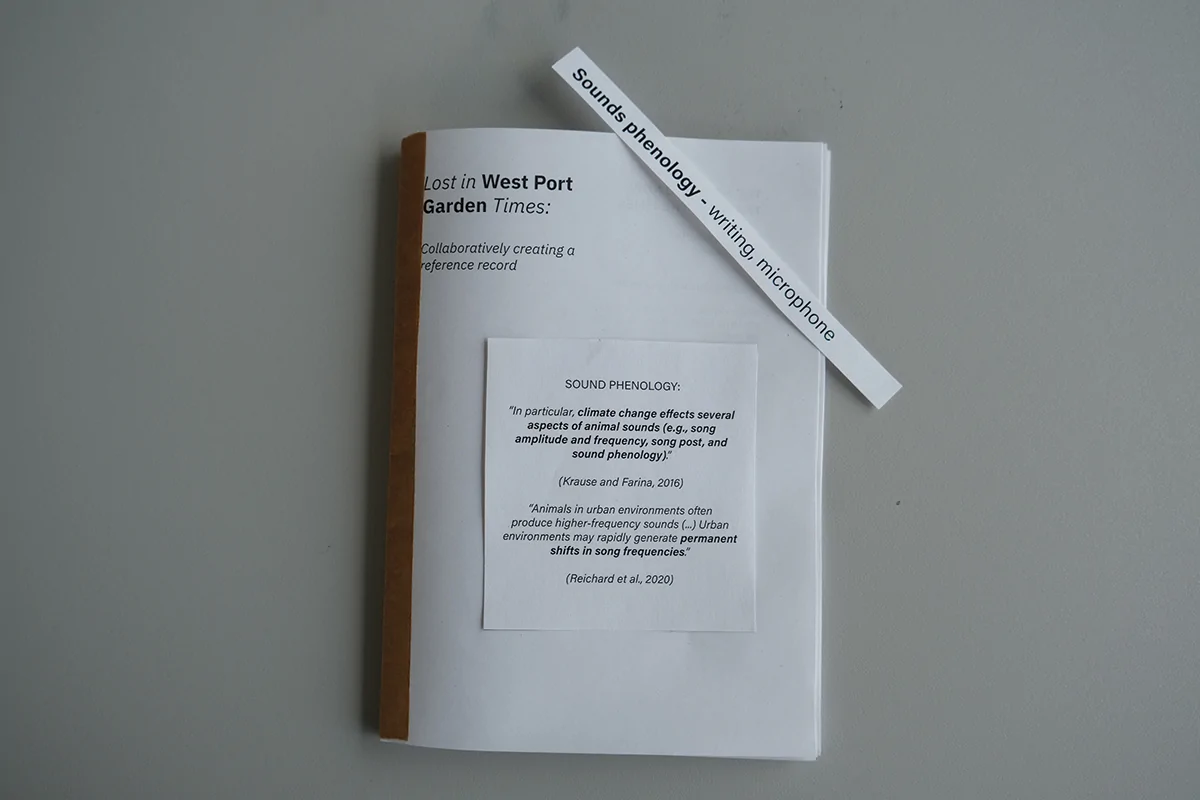
The photo of the workbook for the workshop, along with one prompt for the sound lens on the top. Photograph by the author.

**Figure 4.**
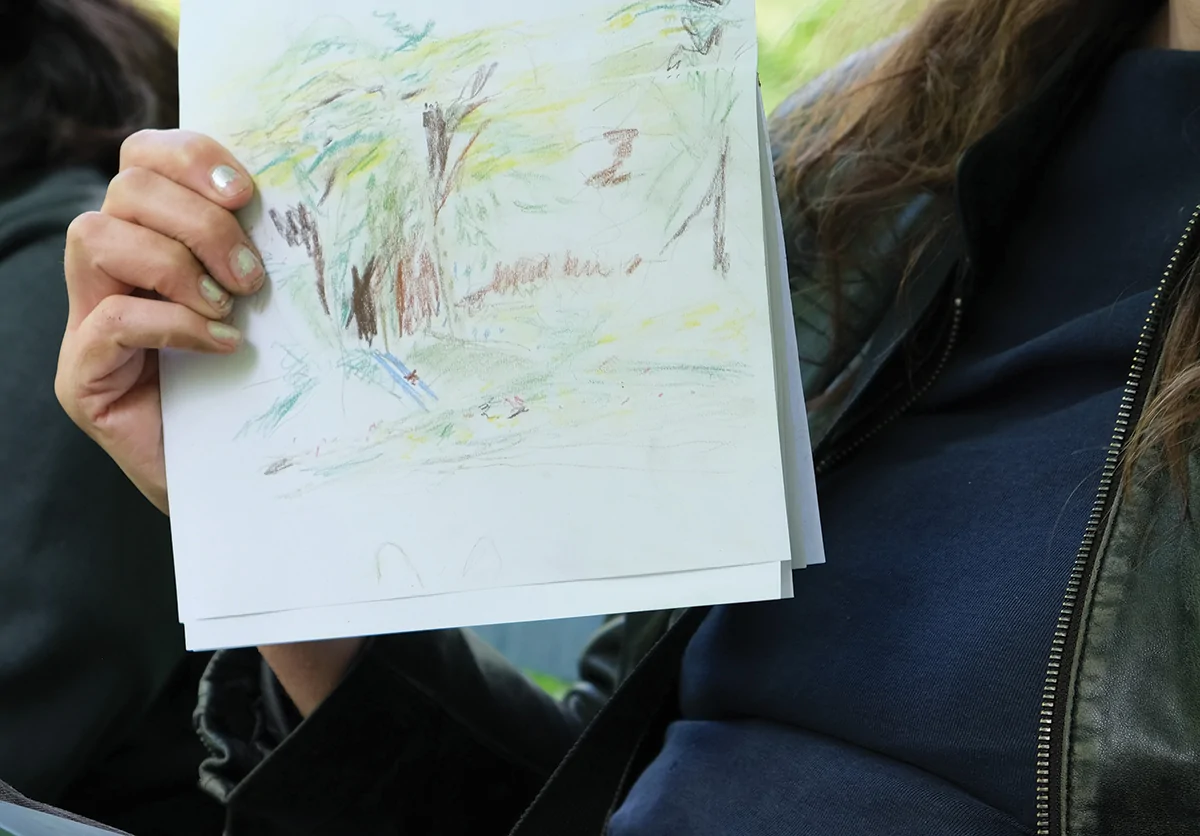
Participants (with the lens of colour) presenting their workbook page. Photograph by the author.

**Figure 5.**
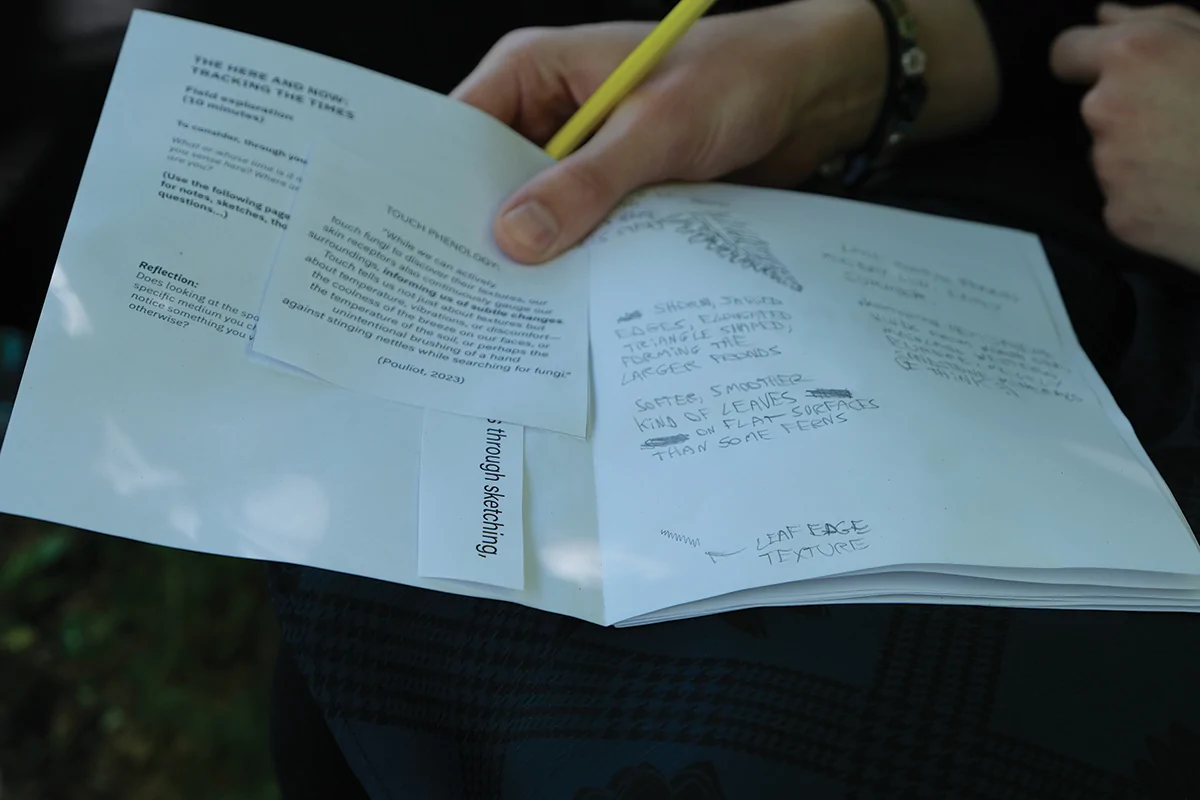
Photo of one of the participants’ workbook pages. Photograph by the author.

For phenological engagement, the workbook included two activities. The first asked the participants to zoom in on an observed phenomenon and create a detailed protocol for what it looks, sounds, or feels like right now. The second asked them to propose a way of noticing this phenomenon next time. The workbook also included phenological guide concepts to help participants’ notice peculiarities in the space (e.g. what perhaps should not be here, what might be missing, or what might be different) and to identify any disturbances that could point to temporal issues. Like the lenses, these concepts were scoped by the author from recent phenological studies discussing temporal issues in ecologies. These concepts were unexpected events (such as insect outbreaks, e.g. Büntgen et al. 2020); advances/delays (such as the early budburst in the UK, e.g. Ffrench-Constant et al. 2016); varied speeds of development (adaption speeds, e.Otto et al. 2018); timing disruptions (e.g. birds stop singing with traffic passing, Monniez et al. 2022); absences (e.g. butterflies absences in urban areas, e.g. Kuussaari et al. 2021); new presences (plants expanding their geographic range under warming, e.g. Lustenhouwer et al. 2018); aseasonality (not all plant and animal behaviour is seasonal, for example, in aseasonal climates, see O’brien et al. 2024).

Phenology thus played a two-fold role in the workshop: (1) providing some catalysing vocabulary for thinking about the changing temporalities, and (2) offering a method for capturing the relational timing through an observation protocol.

The workbooks were collected from participants, and the data were digitised from their protocols. The sharing discussion was audio-recorded. The workbook protocol written data were presented in raw form below (with minor edits for consistency) to share participants’ methods of attunement. The discussion data was analysed interpretively, reading each response in depth while attending to patterns and recurring concerns across participants, to respond to the question: ‘what kind of work did the concepts and lenses do in the workshop?’ Workshop facilitation and subsequent analysis were done by the author.

## Outcomes: participants’ attunement methods from the workbook

To illustrate participants’ thinking during the workshop, Table 1 presents their written protocols for a method of attending to the ‘here and now’ of the garden. Each participant brought further reflections to the discussion about what they noticed and how, and what felt significant to attend to. Some protocols included drawings which are not included.

**Table 1.** Written parts of the participants suggested protocols for observing the phenomena next time.

Participant	Title	Method for next time	Keywords
P1	A way with the birds	To choose a spot, be quite still and take in the overall sounds, such as different bird calls. Focus on specific bird calls. You could try to identify one particular bird, and then follow the calls. Pay attention to where they are going. What are the different plants it might be interacting with? Make connections between the bird call and what else is happening.	Sounds, following bird individuals through sight, interactions
P2	Multimedia meditative recording	Close your eyes, get a sense of the sounds, for roughly one minute, and be aware of your expectations of what you want to hear or expect to hear in a garden. Record with a device. Compare your own experience to recording. What stands out to you, and then what stands out to you, as a listener of the recording? Be aware of your anthropocentric assumptions.	Recording devices, comparisons
P3	Movement directions	To look at the movement changes through the shapes of the plants. Not only does the wind make the shape, but also human and nonhuman interactions (e.g. through eating bits of the leaves, stomping on). Can you see the traces of different wind directions of the past in the garden? To see the other nonhuman interactions and movements, and how everything changes each other’s shapes.	Interactions, directions
P4	Moments of disruptions	Notice how others move around you, how the shadows of the trees and branches move on the wall of the building, how birds jump around the ground. Note specific instances when their behaviour is suddenly disrupted, and what is causing this.	Disruptions, movement
P5	Softness testing	Touching the ferns and other plants and seeing how they feel. How humid/dry or warm/cold they are. Look at the silhouettes and edges of plants and discern texture by sight. At different times of year, it would appear quite different, including on tree bark. Assess how much give there is when you are poking the plant, which could be indicative of how healthy a tree might be (e.g. if bark is falling off).	Health, textures
P6	Squintology	It is called squintology, as you can get a bit overwhelmed by the shades, so if you squint, you get a more distinct focus on the shade. See the light in the dark. Get a more top-down and bottom-up perspective (seeing the garden from the top and then from the bottom of the canopy). To be tuned to this change, the change in the light. Ideally, you would have squintology that covers every second, every minute, every hour, every day, to see the seasons. Get the broad outline of the colour, of shapes, but also get perhaps some sort of details in a very small space, maybe just a leaf.	Light change
P7	Touching and being touched	There are two broad components to this methodology: tracking the process and tracking the intentionality. Tracking the process is about mapping the changes, what did it feel like when you touched a particular thing, or when you were touched by a particular thing. How did it change your perception (e.g. of temperature), or subsequent feelings of change in your body? The second one is mapping whether it’s intentional or not. How did you encounter a particular touch, and how did you feel about it? Do you feel happy about it, or not? And what does it mean for understanding of agency, and for the form of action? Being attentive to this dimension of the agency.	Agency, intentionality, touch
P8	Multiple timescapes of a single plant	Observation of multiple existing timescapes in one plant. Look at a plant (e.g. poppy) and how you could see in one plant, the bud, the bloom. At peak and passing peak bloom, there is an opportunity to catch a glimpse of the red and pink poppies in the garden. There will be various stages of budding, bloom, spent bloom and seed heads. Upon walking through the herbaceous perennial garden, you can witness other plants in similar stages. Imagine interactions below ground that are not visible. Observe interactions above ground which are.	Multiple stages of a plant

## Responses and discussion: attending to the garden space through approaches inspired by phenology

### Temporal interrelations across species categories

The participant protocols reveal a shared orientation towards temporal interconnection, tracing how one thing shapes, disrupts, or depends on another. Participants consistently attended to relations rather than isolated species or phenomena, moving between scales and across species categories in ways that resonate with phenological attention to ecological coordination. The workshop responses demonstrate that phenological approaches can highlight the complex interrelatedness of our dependence on each other, attending to ecological time as always multiple, local, and relational (Bastian 2012; Bastian and Hawitt 2023; Tsing 2015). Phenology, as a concrete study of species timings, can support humanities-based attention to multispecies habitats and agencies by offering tools and approaches to structure and scaffold observation. Reworking these tools through more-than-human scholarship (Haraway 2016; van Dooren 2014) could inform scientific or citizen-science-based phenological approaches.

One such synthesis of phenological mechanisms involved considering new kinds of phenological lenses, incorporating concepts from phenology, and inviting the creation of observation protocols. In the workshop, participants explored relations through the use of alternative phenological lenses, attending to cross-cutting timings and the material agencies of local ecologies (Jones 2008) by tuning attention to the multiple at once, sensing across species. Over the two-hour exploration, the lenses drew participants attention from single expressions in the site towards the relations between them, as one participant reflected, ‘And so then I started to think about the other things in the gardens, such as the insects, and what was in the garden that the birds were here for’ (P1). At times, holding an instrument of listening, even without using it, sharpened the focus, as P1 noted, ‘I didn’t listen to the recording, but I found just the act of doing that sort of framed a period of listening where I was a little bit more focused’ (P1). Such alternative lenses are useful because they cut across species categories. Because sounds, shades, textures, and movements have their own capacities to affect and be affected (Bennett 2010), attending to them lets participants notice forms of agency and relation that a focus on individual organisms would overlook.

Yet the lenses proved broad and participants invented their own ways of narrowing focus. For example, by sitting next to something familiar: ‘When I turned around, I saw a poppy that I have in my garden back at home, but I have never been able to see bloom because I have always been here [in the UK]’ (P8), or by limiting what they could see: ‘You can get a bit overwhelmed by the shades, so if you squint, you get a more distinct focus on the shade’ (P6). These forms of selective attention, of listening by pressing record, squinting, or focusing observations on a familiar flower, were about learning to sense differently and finding appropriate scales of perception, which participants often did quite effortlessly.

Writing and reflecting on the recording protocols extended this practice of attending to interrelations. As noted, the citizen-science recording usually focuses on single species, and its protocols are defined top-down by professional scientists to keep the data accurate and usable for analysis. The humanities participants instead envisioned protocols that explore interconnections between multiple phenomena and cut across species and other taxonomic groups. As participants drafted their own instructions, they began to describe relations rather than isolated observations, often shifting between zoomed-in and zoomed-out perspectives, from the shade of a single leaf to the movement of the canopy above. This often resulted in new realisations, as one participant noted in the discussion, ‘While I was writing the instructions, I realised that not only the wind makes the shape [of plants], but also other human and nonhuman interactions’ (P3). Through writing the protocols, participants mediated between what is there and what seemed important to attend to next time, as a process of assigning importance. These observations are a small fragment of what can be sensed there, unique to that specific morning, and cannot encompass the plurality of happenings actually unfolding. They are conditioned by the workshop’s time constraints, by participants’ prior knowledge and interests, and by the site itself as a human-tended urban garden. The workshop nonetheless demonstrates how humanities-based practices can begin to reveal ecological relations that others can share and take up as starting points for future observation.

### Observation in times of precarity

To understand how coordination happens in times of rapid change, we need conceptual tools that can hold the instability and unevenness of more-than-human worlds. Scholars in the environmental humanities have described more-than-human temporalities as not always fitting easily into human-centred timelines (Van Patter 2023). Gan (2017), for example, discusses the grain of rice through several concepts (e.g. dormancy, recursion, continuity) to suggest other ways of attending to its multiple rhythms and scales, beyond the causality of standardised measures of time (days, years). Gan also notes that expanded conceptualisations of temporality provide ways to address the instability and unravelling of anthropocentric progress, similarly to Tsing (2015), who asks, ‘What if precarity, indeterminacy, and what we imagine as trivial are the center of the systematicity we seek?’ (20). This question shifts our attention from stability to the uneven, often fragile ways that nonhumans have to operate.

Phenological sciences can provide a number of concepts to think with in the humanities, some of which were utilised in the workshop (e.g. absence, delay, disruption). Beyond simply noticing ‘what is there’, applying other conceptual tools can simultaneously open the registers for irregular patterns, the gaps, delays, and disruptions that shape how we live with others. In the workshop, one participant said, ‘I think there is intimacy also in the absences, because I was expecting to hear maybe insects buzzing here at some point. So I’m curious if someone’s here’ (P3), showing an attentiveness to what is missing as much as to what is present. Paying attention to these layered rhythms and being aware of other modes of presence means resisting the urge to generalise or to look for coherence within the seasons, and instead accepting the likelihood of encountering messy and uneven shared worlds. What emerges through such approaches is a polyphony of overlapping rhythms (Tsing 2015), a thickened sense of ecological time.

## Conclusion

On 6 June 2024, eight participants spent two hours in a small Edinburgh garden asking whose time it was. What emerged were eight distinct methods, each shaped by a particular body, lens, and way of attending. A single workshop is only a beginning, but the approach it demonstrates is repeatable and cumulative. The protocols participants developed proposed ways of coming back to the same site, of noticing what had shifted since the last visit, as phenological observation has always depended on records built across years and decades. Humanities-based approaches can widen who takes part in this long-term noticing, and can shift what that noticing attends to, moving beyond single species to the relationships between them. For those outside scientific institutions, lenses, concepts, and protocols reworked in this paper can provide entry points into this way of attending. As ecological change continues to reshape multispecies relations, the capacity to notice, record, and interpret what is shifting in a local place becomes a cultural concern as much as a scientific one.
